# Supplementary material for: Over expression of modified Isomaltulose Synthase Gene II (ImSyGII) under single and double promoters drive unprecedented sugar contents in sugarcane
Source: PLoS One. 2024 Nov 19;19(11):e0311797. doi: 10.1371/journal.pone.0311797 (PMC11575802; doi:10.1371/journal.pone.0311797)

**Supplementary materials**

**Descriptive Statistics of Raw Data**

**Descriptive Statistics for Figure S-7A**

**Stem Leaf**

N 12 12

Sum 16.400 25.700

Mean 1.3667 2.1417

SD 0.4849 0.8174

Variance 0.2352 0.6681

SE Mean 0.1400 0.2360

C.V. 35.482 38.165

Minimum 0.0000 0.0000

Maximum 1.8000 2.7000

Biased Var 0.2156 0.6124

**Descriptive Statistics of Data in figure S-7B**

**Stem Leaf**

N 12 12

Sum 21.200 36.300

Mean 1.7667 3.0250

SD 0.6527 1.2129

Variance 0.4261 1.4711

SE Mean 0.1884 0.3501

C.V. 36.947 40.096

Minimum 0.0000 0.0000

Maximum 2.3000 3.8000

Biased Var 0.3906 1.3485

**Descriptive Statistics of Data in Figure S-7C**

**Stem Leaf**

N 14 14

Sum 45.900 21.300

Mean 3.2786 1.5214

SD 1.3215 0.5951

Variance 1.7464 0.3541

SE Mean 0.3532 0.1590

C.V. 40.308 39.113

Minimum 0.0000 0.0000

Maximum 4.8000 2.4000

Biased Var 1.6217 0.3288

**Descriptive Statistics of Data in figure S-7D**

**Stem Leaf**

N 14 14

Sum 56.100 33.700

Mean 4.0071 2.4071

SD 1.5701 0.9008

Variance 2.4653 0.8115

SE Mean 0.4196 0.2408

C.V. 39.183 37.423

Minimum 0.0000 0.0000

Maximum 5.8000 3.4000

Biased Var 2.2892 0.7535

**Descriptive Statistics of Data in figure S-8A**

**V002 V003**

N 14 14

Sum 1503.5 830.85

Mean 107.39 59.346

SD 50.299 28.537

Variance 2530.0 814.35

SE Mean 13.443 7.6268

C.V. 46.837 48.085

Minimum 0.0000 0.0000

Maximum 169.23 91.000

Biased Var 2349.2 756.18

**Descriptive Statistics of data in figure S-8B**

**Increase increa~01**

N 12 12

Sum 618.30 756.40

Mean 51.525 63.033

SD 24.392 32.335

Variance 594.95 1045.5

SE Mean 7.0413 9.3342

C.V. 47.339 51.297

Minimum 0.0000 0.0000

Maximum 68.000 105.00

Biased Var 545.37 958.39

**Descriptive Statistics of Figure S-9A**

**Leaf Stalk**

N 13 13

Sum 29.200 41.200

Mean 2.2462 3.1692

SD 0.8333 1.2106

Variance 0.6944 1.4656

SE Mean 0.2311 0.3358

C.V. 37.098 38.200

Minimum 0.0000 0.0000

Maximum 2.9000 4.0000

Biased Var 0.6409 1.3529

**Descriptive Statistics of Figure S-9B**

**Leaf Stalk**

N 13 13

Sum 43.800 56.900

Mean 3.3692 4.3769

SD 1.3054 1.7470

Variance 1.7040 3.0519

SE Mean 0.3620 0.4845

C.V. 38.744 39.913

Minimum 0.0000 0.0000

Maximum 4.3000 5.5000

Biased Var 1.5729 2.8172

**Descriptive Statistics of Figure S-9C**

**Descriptive Statistics**

**variation Variat~01**

N 13 13

Sum 488.50 342.50

Mean 37.577 26.346

SD 19.598 12.559

Variance 384.10 157.72

SE Mean 5.4356 3.4832

C.V. 52.156 47.668

Minimum 0.0000 0.0000

Maximum 62.000 38.400

Biased Var 354.55 145.59

**Descriptive Statistics OF Data in figure S-9D**

**Variation variat~01**

N 12 12

Sum 318.97 415.80

Mean 26.581 34.650

SD 12.676 17.280

Variance 160.69 298.60

SE Mean 3.6593 4.9884

C.V. 47.689 49.871

Minimum 0.0000 0.0000

Maximum 35.900 55.000

Biased Var 147.29 273.72

**Descriptive Statistics of Data in figure S-10A**

**Leaf Stalk**

N 12 12

Sum 41.800 54.800

Mean 3.4833 4.5667

SD 1.4237 1.9242

Variance 2.0270 3.7024

SE Mean 0.4110 0.5555

C.V. 40.872 42.135

Minimum 0.0000 0.0000

Maximum 4.5000 5.8000

Biased Var 1.8581 3.3939

**Descriptive Statistics of Figure S-10B**

**Leaf Stalk**

N 11 11

Sum 45.400 63.700

Mean 4.1273 5.7909

SD 1.0650 1.5990

Variance 1.1342 2.5569

SE Mean 0.3211 0.4821

C.V. 25.803 27.613

Minimum 1.0000 1.0000

Maximum 4.8000 6.7000

Biased Var 1.0311 2.3245

**Descriptive Statistics of Figure S-11**

**Variable N Mean SD Minimum Maximum**

glucose 56 91.464 16.936 65.000 127.00

fructose 56 67.571 16.696 43.000 98.000

sucrose 56 190.61 37.030 120.00 287.00

isomaltul 56 421.93 61.263 190.00 489.00

trehalulo 56 91.679 16.570 66.000 127.00

**Descriptive Statistics of data in figure S-12A**

**glucose sucrose fructose isomaltul trehalulo**

N 13 13 13 13 13

Sum 915 1955 594 4814 942

Mean 70.385 150.38 45.692 370.31 72.462

SD 3.2542 26.066 0.8549 91.798 6.1049

Variance 10.590 679.42 0.7308 8426.9 37.269

SE Mean 0.9025 7.2293 0.2371 25.460 1.6932

C.V. 4.6234 17.333 1.8709 24.790 8.4250

Minimum 65.000 120.00 43.000 190.00 66.000

Maximum 76.000 195.00 46.000 465.00 87.000

Biased Var 9.7751 627.16 0.6746 7778.7 34.402

**Descriptive Statistics of Data in figure S-12B**

**Glucose Sucrose Fructose Isomaltul Trehalulo**

N 13 13 13 13 13

Sum 1128 2345 822 4732 1062

Mean 86.769 180.38 63.231 364.00 81.692

SD 5.9041 22.559 8.0224 90.161 4.2892

Variance 34.859 508.92 64.359 8129.0 18.397

SE Mean 1.6375 6.2568 2.2250 25.006 1.1896

C.V. 6.8044 12.506 12.688 24.769 5.2505

Minimum 76.000 145.00 54.000 221.00 74.000

Maximum 97.000 220.00 78.000 488.00 87.000

Biased Var 32.178 469.78 59.408 7503.7 16.982

**Descriptive Statistics of data in figure S-12C**

**Glucose Sucrose Fructose Isomaltul**

N 15 15 15 15

Sum 1403 2910 1064 6364

Mean 93.533 194.00 70.933 424.27

SD 9.1875 16.270 9.0196 84.364

Variance 84.410 264.71 81.352 7117.4

SE Mean 2.3722 4.2009 2.3288 21.783

C.V. 9.8227 8.3866 12.716 19.885

Minimum 83.000 175.00 59.000 259.00

Maximum 112.00 235.00 89.000 527.00

Biased Var 78.782 247.07 75.929 6642.9

**Descriptive Statistics of data in figure 12D**

**Descriptive Statistics**

**glucose sucrose fructose isomaltul trehalulo**

N 15 15 15 15 15

Sum 1676 3464 1304 7769 1677

Mean 111.73 230.93 86.933 517.93 111.80

SD 11.209 27.380 9.0669 113.43 10.435

Variance 125.64 749.64 82.210 12867 108.89

SE Mean 2.8941 7.0694 2.3411 29.288 2.6943

C.V. 10.032 11.856 10.430 21.901 9.3335

Minimum 97.000 204.00 73.000 318.00 95.000

Maximum 127.00 287.00 98.000 648.00 127.00

Biased Var 117.26 699.66 76.729 12009 101.63

**Descriptive Statistics of data in figure 12E**

**Glucose Sucrose Fructose Isomaltul**

N 14 14 14 14

Sum 1087 2136 848 0

Mean 77.643 152.57 60.571 0.0000

SD 17.788 36.901 17.814 0.0000

Variance 316.40 1361.6 317.34 0.0000

SE Mean 4.7540 9.8621 4.7610 0.0000

C.V. 22.910 24.186 29.410 M

Minimum 45.000 85.000 27.000 0.0000

Maximum 106.00 198.00 82.000 0.0000

Biased Var 293.80 1264.4 294.67 0.0000

**Figure S-13: Southern Blotting Results**


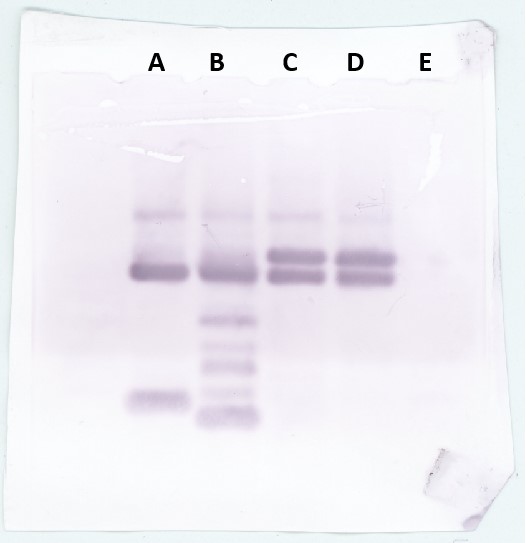

Supplement: S1 File — (DOCX) [file pone.0311797.s001.docx]
