# Supplementary figures and images for: Over expression of modified Isomaltulose Synthase Gene II (ImSyGII) under single and double promoters drive unprecedented sugar contents in sugarcane
Source: PLoS One. 2024 Nov 19;19(11):e0311797. doi: 10.1371/journal.pone.0311797 (PMC11575802; doi:10.1371/journal.pone.0311797)

**S1_RAW_GEL IMAGES”**

**Supplementary Gel Pictures**

**S-2A**

**
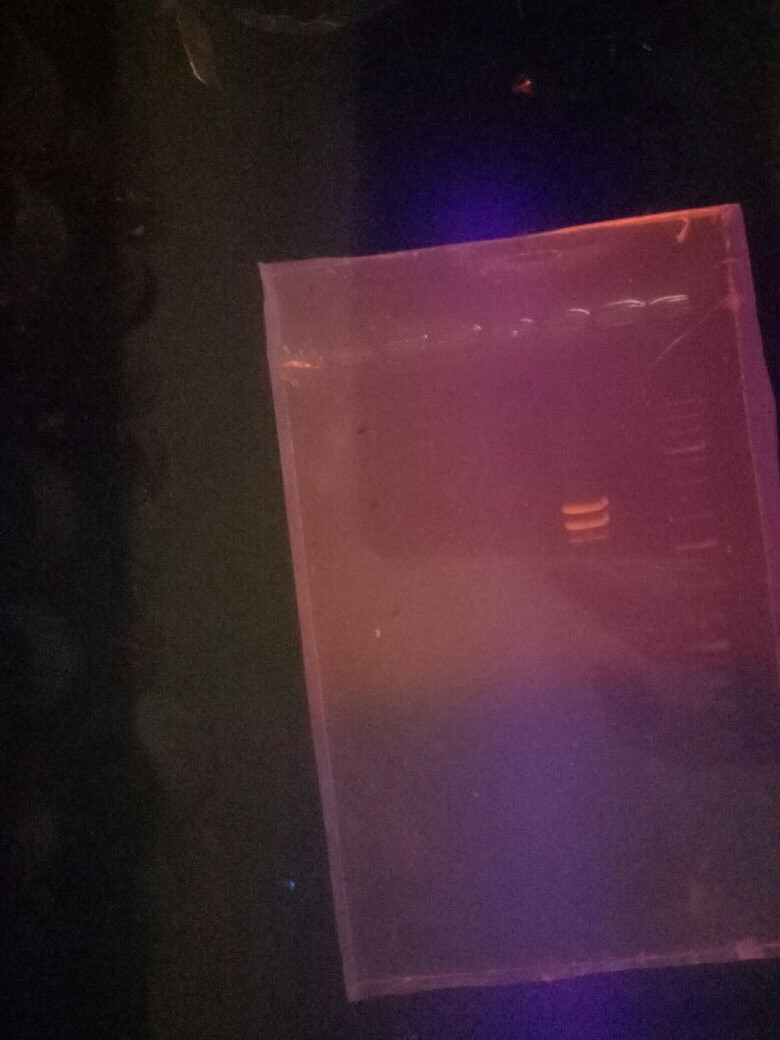
**

**
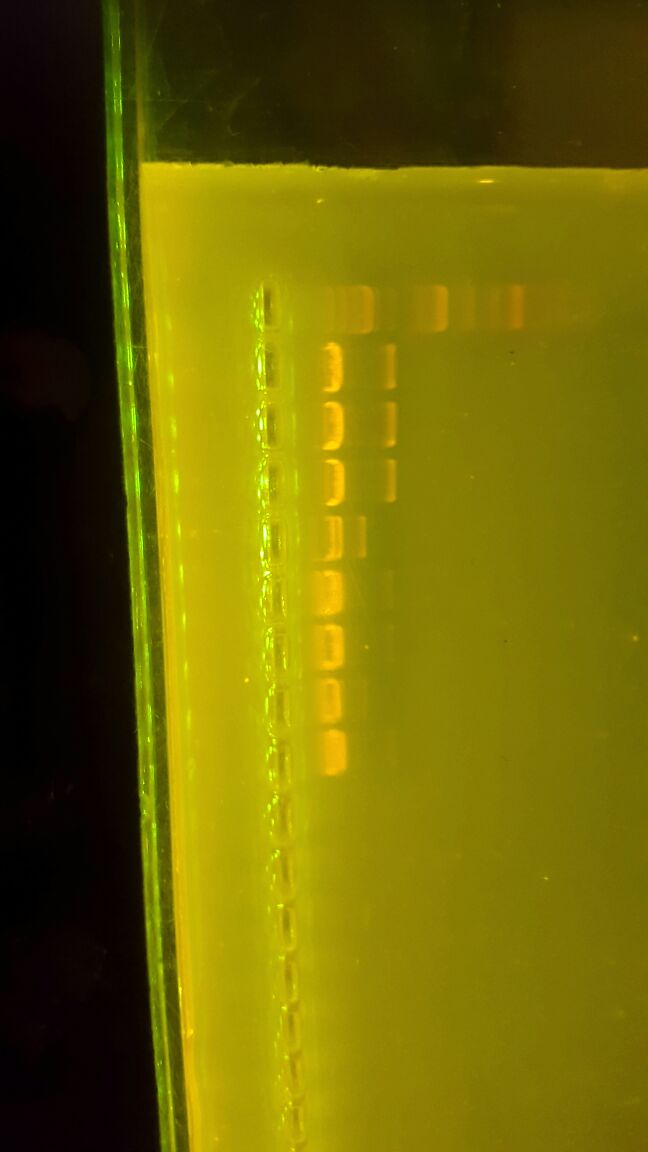
**

**S-2C**

**
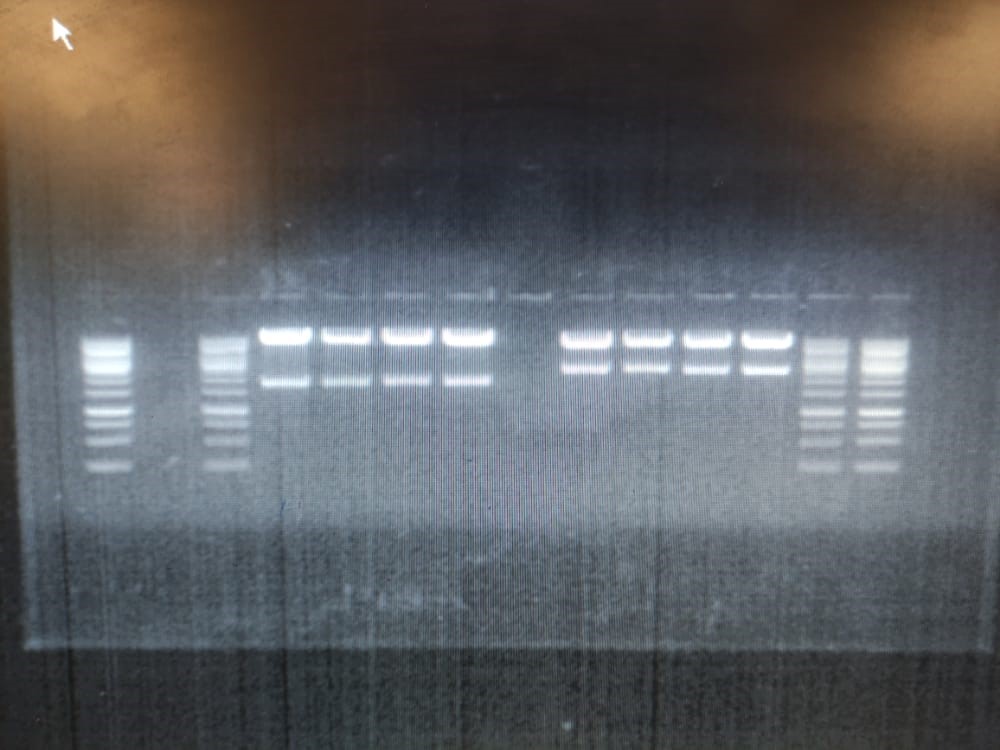
**

**S-2D**

**
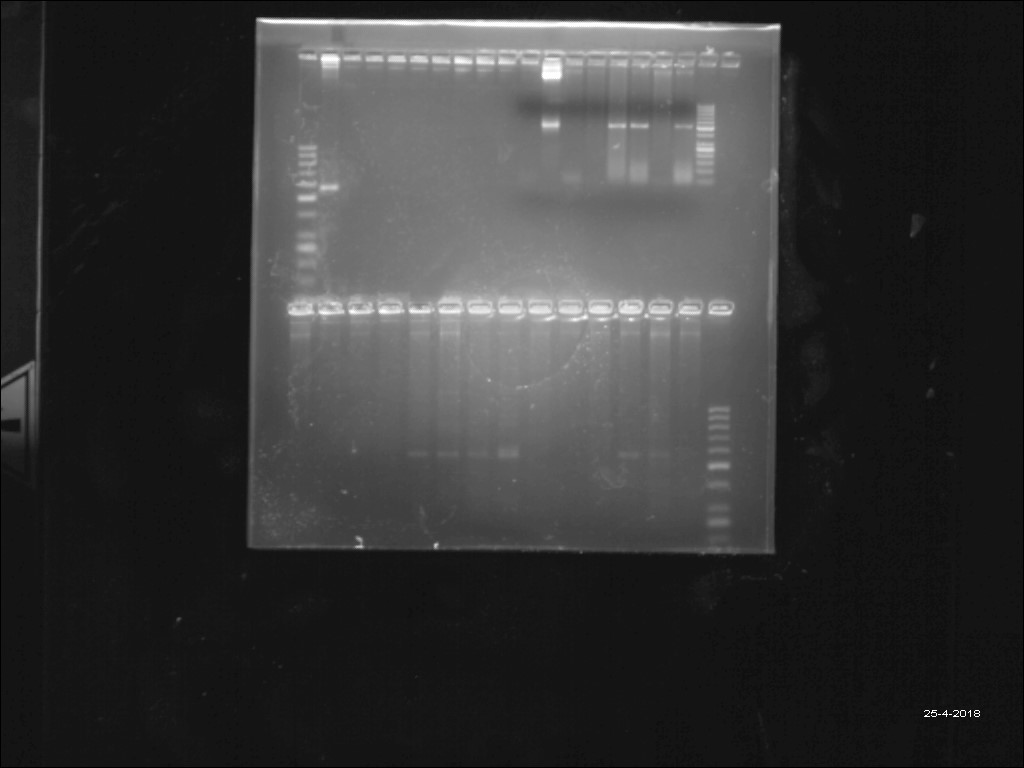
**

Supplement: S1 Raw images — (DOCX) [file pone.0311797.s002.docx]
