# Supplementary material for: Over expression of modified Isomaltulose Synthase Gene II (ImSyGII) under single and double promoters drive unprecedented sugar contents in sugarcane
Source: PLoS One. 2024 Nov 19;19(11):e0311797. doi: 10.1371/journal.pone.0311797 (PMC11575802; doi:10.1371/journal.pone.0311797)
Supplement: Attachment — Submitted filename: review-plos-one.docx [file pone.0311797.s003.docx]

**Over expression of Modified Isomaltulose Synthase Gene II (ImSyGII) Under Single and Double Promoters Drive Unprecedented Sugar Contents in Sugarcane**

This research topic is very important for an agricultural country whose sugar production is majorly relying on sugarcane.

- Abstract: Language needs improvement. It’s not very understandable in the first read.
- Which promoter gene combinations were introduced. Either different leaf and stalk specific promoters were used or only one promoter?
- The way author mentioned the cloning and transformation strategy is very ambiguous.
- Which sugarcane genotype was transformed?
- Headings pattern is not uniform throughout the manuscript
- What was the original source of ImSyGII gene?
- Graphical representation of vectors should be improved. Mention the size of each vector.
- In the description of genetic maps, sense of explanation is destroyed in the effort to remove plagiarism.
- Methodology is very poorly written.
- sodium salt 1M NaCl? Recheck your protocol.
- What is the purpose of 6x blue dye staining in coating the gold particles.
- Author should recheck the protocols and their explanation in the manuscript.
