# Supplementary material for: Over expression of modified Isomaltulose Synthase Gene II (ImSyGII) under single and double promoters drive unprecedented sugar contents in sugarcane
Source: PLoS One. 2024 Nov 19;19(11):e0311797. doi: 10.1371/journal.pone.0311797 (PMC11575802; doi:10.1371/journal.pone.0311797)
Supplement: Attachment — Submitted filename: The Answers to the editorial and Reviewer.docx for Plos one.docx [file pone.0311797.s004.docx]

**A Rebuttal Letter**

**The Answers to the editorial and Reviewer’s Comments**

PONE-D-23-31445

”**Over expression of Modified Isomaltulose Synthase Gene II (ImSyGII) Under Single and Double Promoters Drive Unprecedented Sugar Contents in Sugarcane”**
PLOS ONE

**Answers to the Additional Editor Comments :** 

Over expression of Modified Isomaltulose Synthase Gene II (ImSyGII) Under Single and Double Promoters Drive Unprecedented Sugar Contents in Sugarcane

This research topic is very important for an agricultural country whose sugar production is majorly relying on sugarcane.
•1. Abstract: Language needs improvement. It’s not very understandable in the first read.

**Answer 1:** The abstract is made more understandable and language improvement has been done considerably.

2. Which promoter gene combinations were introduced. Either different leaf and stalk specific promoters were used or only one promoter?

**Answer 2:**

It was written in the manuscript (page number 4, line 6) in the materials and method section that two different promoters were used in three different promoter combinations. One was leaf specific while other was stem specific while in the third combination both were combined in a single plant expression vector.

3. The way author mentioned the cloning and transformation strategy is very ambiguous.

**Answer 3:**

The cloning and transformation strategy was described in a very detailed manner. Further clarity was also done after careful revisions.

4. Which sugarcane genotype was transformed?

**Answer 4:** The page 5 (line no. 1) of materials and method section shows that sugarcane genotype HSF-240 was used for transformation. Similarly, in many subsequent pages the name of sugarcane genotype was mentioned time and again.

5. Headings pattern is not uniform throughout the manuscript

**Answer 5:** Heading pattern is also made uniformed throughout the manuscript.

6. What was the original source of ImSyGII gene?

**Answer 6:** The original source of the ImSyGII was bacterial strain *Pentoea dispersa* as described in the manuscript.

7. Graphical representation of vectors should be improved. Mention the size of each vector.

**Answer 7:** The size of the constructs with gene fragment sizes are given in the materials and method section and in maps figure no. 1A.B.C respectively.

8. In the description of genetic maps, sense of explanation is destroyed in the effort to remove plagiarism.

**Answer 8:**

In the description of genetic maps, all figures have been explained in a very detailed way. Figures description does not need any change to avoid plagiarism because already similarity index of the manuscript is very low and figures description have nothing to do with plagiarism.

9. Methodology is very poorly written.

**Answer 9:** Methodology is improved as suggested.

10. sodium salt 1M NaCl? Recheck your protocol.

**Answer 10**: High salt concentration is necessary to avoid any possible degradation moreover to create high alkaline conditions therefore salt is used . The adopted protocol was rechecked.

11. What is the purpose of 6x blue dye staining in coating the gold particles.

**Answer 11:**

I have not used gold particles while used tungsten particles as described in (page 6 line 2) the submitted manuscript. Secondly, 6x blue dye staining was done to indicate the position of bombarded materials into the sugarcane callus.

12. Author should recheck the protocols and their explanation in the manuscript.

**Answer 12:**  Authors have checked the protocols and their explanation was made in a very described way in the manuscript. Moreover, in case of any specific indication it can be changed accordingly.

5. **Review Comments to the Author**

Please use the space provided to explain your answers to the questions above. You may also include additional comments for the author, including concerns about dual publication, research ethics, or publication ethics. (Please upload your review as an attachment if it exceeds 20,000 characters)

**Comments of Reviewer #1 and Answers:**

The manuscript reports expression of isomaltulose synthetic genes in sugarcane through a three gene constructs driven under promoters Zea mays ubiquitin and cestrum yellow leaf curl virus in the single and double combined stacked system. The authors report that employment of three gene constructs collectively produced elite sugar lines producing more than 78% enhancements in whole sugar recovery percentage. They also report that mature internode is highly efficient and receptive for the production of isomaltulose. Overall, they found an increased Brix/ sugar in the transgenic sugarcane lines than control lines. This is a novel finding with wonderful achievement in scientific research and merits publication.
However the authors have to clarify on the comments indicated in the manuscript.

1. Cane yield is an important economic parameter in transgenic lines , but the manuscript is silent on that. Let the authors clarify.

**Answer 1:** The present work has used the scientific strategy to enhance the sugar recovery percentage of the sugarcane which will ultimately enhance the sugar yield.
2. However isomaltulose is beneficial to human consumption and how it is going to be economically useful?

**Answer 2:** Isomaltulose can be extracted, purified and can be used as an artificial sweetener in our industries like jam jellies and other food products.

3. Is it possible to use such genes in other crops like sugarbeet to improve sugar contents.

**Answer 3:** Yes, it is possible to use these genes in other food crops like sugarbeet. Even, we can enhance the sugar contents of fruits by employing these genes.

4. What is the recovery percentage of the transgenic lines as against the control line?

**Answer 4:** The transgenic line in our study produced 21% sugar recovery as in TCTE lines than control (10%).

5. In your findings the role of stem specific promoters were investigated to enhance the production of sugars in transgenic sugarcane. What is the impact of leaf specific promoters in transgenic sugarcane?

**Answer 5:** The leaf specific promoters may enhance the vegetative growth and can show good gene product at leaves which are not required in the present study while stem specific promoters will enhance the gene activity at stems conclusively adding other phenotypic features in transgenic stems as described in our study.

6. What is the impact of your study on other features of plants?

**Answer 6:** The plants may have high photosynthetic efficiency and can produce better sugar signaling mechanism than control lines.

7. Do control lines have endogenous ImSyGII gene?

**Answer 7:** No, the control lines don’t have any ImSyGII gene.

**Answers to the reviewer 2 comments**

Reviewer #2: This research topic is very important for an agricultural country whose sugar production is majorly relying on sugarcane.

1. The whole manuscript should be written again.

**Answer 1:** The whole manuscript is proofread and improved accordingly with necessary corrections.

2. Abstract: Language needs improvement. It’s not very understandable in the first read.

**Answer 2:** The language of the abstract is made clear and understandable. It is written in a very scientific manner.

3. Which promoter gene combinations were introduced. Either different leaf and stalk specific promoters were used or only one promoter?

**Answer 3:**

It was written very clearly in my manuscript (page number 4, line 6) in the materials and method section that two different promoters were used in three different combinations. One was leaf specific while other was stem specific while in the third combination both were combined in a single plant expression vector. The STE1 was used for expression in leaves while STE2 was used for expression of ImSyGII in stem.

4. Which sugarcane genotype was transformed? Mention it in the abstract.

**Answer 4:** The page 5 (line no. 1) of materials and method section shows that sugarcane genotype HSF-240 was used for transformation. Similarly, in many subsequent pages the name of sugarcane genotype was mentioned time and again. I will add it in the abstract section.

5. Headings pattern is not uniform throughout the manuscript.

**Answer 5:** Heading pattern is made uniform throughout the manuscript and if there is any ambiguity, please mention clearly. I will change it accordingly.

6. What was the original source of ImSyGII gene?

**Answer 6:** The original source of the ImSyGII was bacterial strain *Pentoea dispersa* as described.

7. Graphical representation of vectors should be improved. Mention the size of each vector.In the description of genetic maps, sense of explanation is destroyed in the effort to remove plagiarism.

**Answer 7:**

The graphical representation of the vectors can be improved as advised. In the description of genetic maps, all figures have been explained in a very detailed way. If editor needs any specific details it can be added. Figures description does not need any change to avoid plagiarism because already similarity index of the manuscript is very low and figures description have nothing to do with plagiarism.

**Answer 8:** The size of the constructs with gene fragment sizes are given in the materials and method section and in maps figure no. 1A.B.C respectively.

9. Methodology is very poorly written.

**Answer 9:** Methodology was written in a very detailed way without any grammatical errors. However, it is further Improved. If you find any error, it must be mentioned specifically.

10. sodium salt 1M NaCl? Recheck your protocol.

**Answer 10**: Dear reviewer 2, high salt concentration is necessary to avoid any possible degradation moreover to create high alkaline conditions therefore salt is used . The adopted protocol was rechecked.

11. What is the purpose of 6x blue dye staining in coating the gold particles.

**Answer 11**: I have not used gold particles while used tungsten particles as described in (page 6 line 2) the submitted manuscript. Secondly, 6x blue dye staining was done to indicate the position of bombarded materials into the sugarcane callus.

12. Do the statistical analysis and explain the significant results in your graphs.

**Answer 12**: All the figures mentioned and graphs showed error bars show respective standard deviation of any result. If you want any specific statistical analysis please do mention I have already provided further statistical analysis as supplementary file.
