# Supplementary material for: Over expression of modified Isomaltulose Synthase Gene II (ImSyGII) under single and double promoters drive unprecedented sugar contents in sugarcane
Source: PLoS One. 2024 Nov 19;19(11):e0311797. doi: 10.1371/journal.pone.0311797 (PMC11575802; doi:10.1371/journal.pone.0311797)
Supplement: Attachment — Submitted filename: Comments for Reviewer.docx PLOS one.docx [file pone.0311797.s006.docx]

**Comments for Reviewer:**

The research work seems to be interesting and valuable and conducted in a very detailed manner. The authors have mentioned the protocols and material and methods in a detail. The results were interpreted nicely. However, to enhance the clarity and readability of the manuscript, respond the following questions:

1. Add one to two introductory lines in abstract.
2. Is Sucrose isomerase present endogenously in sugarcane?
3. How the screening of putative transgenic plants were made at initial level?
4. Can this approach be applicable in other food or sugar crops?
5. What size of transgene (ImSyGII) was cloned in plant expression vector.
6. Which sugarcane cultivar was used as a control plant?
7. Is there any chance of PTGS in sugarcane against this transgene when it integrated in sugarcane genome?
8. At which age the callus of sugarcane was in highly receptive stage of transformation.
9. The direct method of transformation was used in this research work, however some of the researchers have also used agrobacterium mediated transformation for monocots do you think that gene gun methods is still a preferred method for monocots?
10. What was the annealing temperature of PCR primers used in this study?
11. What is the way forward of your research work.
