# Supplementary material for: Over expression of modified Isomaltulose Synthase Gene II (ImSyGII) under single and double promoters drive unprecedented sugar contents in sugarcane
Source: PLoS One. 2024 Nov 19;19(11):e0311797. doi: 10.1371/journal.pone.0311797 (PMC11575802; doi:10.1371/journal.pone.0311797)
Supplement: Attachment — Submitted filename: Reviewer comment to authors.pdf [file pone.0311797.s005.pdf]

Reviewer comment to authors

Production of isomaltoluse (IM) that more healthy and sweeter with low lower glycemic index than sucrose is important research for the industry. This manuscript was directed to enhance the content of IM in transgenic sugarcane. The strategy to increase IM was overexpression of isomaltulose synthase gene II (ImSyGII) under single, double, and triple promoter. The authors claimed that the employment of three gene constructs produced elite sugarcane transgenic lines. However, the authors have to clarify bellow comments.

1. It is not clear the reason behind to use single, double, and triple promoter, because the promoter that used in the experiment both Zm-pUbi and pCmYLCV are constitutive promoters and should be expressed in all tissue. But the results showed that Zm-pUbi efficiently expressed in leave and pCmYLCV in stalk.
2. Fig. 1 Illustration of the genetic map should be completed with restriction sites. The figure should also explain whether the Zm-pUbi or pCmYLCV located in the upstream or downstream in the double promoter construct. There is GUS reported gene in the construct, why the experiment did not employ the gene to decide localization of gene expression.
3. Method for screening of putative transgenic was difficult to understand. How many incubations (subculture) in the selection media? Is that true only two times? And how many percentages of transformation efficiency were not presented. Furthermore, how to differentiate the putative transformant that containing triple stacked construct during screening, since all DNA constructs containing same antibiotic resistance, hygromycin. What is the control plant in the last sentence of 2.3. section.
4. PCR amplification of IMSyGII gene was used for screening of transgenic plants, but there was no figure presenting the electrophoresis of the PCR product. Is that true that gel electrophoresis was performed to separate amplicons using 2.5% agarose instead of 1%?
5. Section 2.5. Estimation of copy number by real time PCR. What is the purpose of this section, to identify of the DNA copy number instead of Southern Blot analysis? If the section was proposed to identify the gene copy number, the Southern Blot analysis have to be conducted. However, if the section was directed to analysis the gene expression levels using RT-PCR, the experiment should use RNA. There is no sentence explaining RNA isolation method. In addition, there was no nucleotide primers sequence for qRT-PCR analysis of ImSYGTII gene and beta-actin gene presented in this paper.
6. Section 3.1. (Results), Fig 2 (ABCD) should be completed with the used restriction enzymes. The figures (2ABC) were also unclear, the digestion resulted a clear 2 DNA bands, but there was no DNA band for the rest DNA vector after digestion?
7. Fig 3 and 4, they should be removed from the paper because only presenting the survive callus and plantlet. What about the died materials? This is important data in order to calculate transformation efficiency.
8. Figure 6, Why the transgenic plants were cultivated in open environment? The transgenic plants are prohibited to cultivated in the oven environment (field) before biosafety certifications for community acceptance. Fig 6 was not presented properly as the experiment, and just presented as cropping image. In addition, the authors should consider the effect of environment on the gene expression since the plants were grown in oven environment.
9. Fig. 7, 8, 9 10, what is the unit of expression levels? And please be consistent in using the words stem, stalk or culm.
10. Fig 7 – 10 were the expression levels and Fig 12 is distribution of sugar contents. The expression was increased as well as IM contents, but there no prove on isomaltulose

synthase activity? It is better to include the activity in the paper since the increase in gene expression levels are not always accompanied by the increase of the metabolite.

11. The manuscript mentioned Fig 13, but in fact the Fig 13 is not found.
12. The English is difficult to understand, need revise in the English editing services. There are some mistyping in the manuscripts that should be revised.
